# Supplementary material for: Translation, Adaptation and Validation of the Five-Word Test (Test Delle 5 Parole, T5P) in an Italian Sample: A Rapid Screening for the Assessment of Memory Impairment
Source: Geriatrics (Basel). 2022 Apr 15;7(2):49. doi: 10.3390/geriatrics7020049 (PMC9032514; doi:10.3390/geriatrics7020049)
Supplement: Supplementary file 1 [file geriatrics-07-00049-s001.zip › geriatrics-1634862-supplementary.pdf]

TEST DELLE 5 PAROLE (T5P)

Administration instructions

A – CODING / LEARNING

List Presentation: Show the list of 5 words written on the paper to the subject and say "*Read this list of words aloud and try to remember them, as I will request them shortly*". Once the subject has read the list, keeping it visible, ask the subject "*Can you tell me, still looking at the paper, what is the name of: [below, the semantic suggestions to provide to the subject] the drink, the kitchen utensil, the vehicle, the building and the insect?*"

B - IMMEDIATE RECALL (IR)

Quickly turn the paper over and ask the subject "*Can you tell me the words you just read?*" For the word(s) that is/are not remembered spontaneously (and only for these) ask the patient "*What was the name of the...?*" providing the corresponding semantic suggestion. The examiner counts the number of words correctly recalled (spontaneously and after suggestion): **IMMEDIATE RECALL SCORE**.
1. If the score obtained in the immediate recall (spontaneously and after suggestion) is **equal to 5**, the recording of the words has been effective and you can pass to the delay recall memory task, after an interfering attention test.
2. If the score obtained in the immediate recall is **less than 5**, turn the page and ask the subject to provide the unremembered word (s) in response to the corresponding semantic categorization indices (the aim is to ensure that the subject has indeed registered all the words before evaluating delay memory task).

C - ATTENTIVE INTERFERENT TASK

The aim is to divert the attention of the subject for a sufficient time (ranging from 3 to 5 minutes). The interval can be used to study the spatial orientation-time, to administer a test of calculation in mind, of serial organization of numbers, the test of the design of a clock or the room plan.

D - DELAYED RECALL (DR)

After the interference test, ask the subject "*Could you tell me the five words you read earlier*?" For the word(s) not remembered (and only for this) ask " *What was the name of the...?"* providing the corresponding semantic cue as help. Count the number of words correctly recalled (spontaneously or with cue): **DELAYED RECALL SCORE**.

TOTAL SCORE = IMMEDIATE RECALL SCORE + DELAYED RECALL SCORE

TEST DELLE 5 PAROLE (T5P)

Istruzioni di somministrazione

A – CODIFICA / APPRENDIMENTO

Presentazione della lista: mostrare la lista di 5 parole scritte sul foglio al soggetto e dire "*Legga questa lista di parole ad alta voce e cerchi di ricordarsele, poiché gliele richiederò tra poco*". Una volta che il soggetto ha letto la lista, mantenendola visibile, chiedere al soggetto "*Può dirmi, sempre guardando il foglio, qual è il nome di: [seguono i suggerimenti semantici da fornire al soggetto] la bevanda, l'utensile da cucina, il veicolo, l'edificio e l'insetto?*"

B - RIEVOCAZIONE IMMEDIATA

Voltare rapidamente il foglio e domandare al soggetto *"Può dirmi le parole che ha appena letto?"* Per la/e parola/e che non viene/vengono ricordata/e spontaneamente (e solo per queste) domandare al paziente *"Qual era il nome del...?"* fornendo il suggerimento semantico corrispondente. L'esaminatore conta il numero delle parole correttamente rievocate (spontaneamente e dopo suggerimento): ***PUNTEGGIO DI RIEVOCAZIONE IMMEDIATA (RI).***

1. Se il punteggio ottenuto nella rievocazione immediata (spontaneamente o dopo suggerimento) è **uguale a 5**, la registrazione delle parole è stata efficace e si può passare alla prova di memoria differita, dopo una prova attentiva interferente.
2. Se il punteggio ottenuto nella rievocazione immediata è **inferiore a 5**, rigirare la pagina e quindi chiedere al soggetto di fornire la/le parola/e non ricordata/e in risposta agli indici di categorizzazione semantica corrispondenti (lo scopo è assicurarsi che il soggetto abbia davvero registrato tutte le parole prima di valutare la prova di memoria differita).

C - COMPITO ATTENTIVO INTERFERENTE

Lo scopo è distogliere l'attenzione del soggetto per un tempo sufficiente (da 3 a 5 minuti). L'intervallo può essere sfruttato per studiare l'orientamento spazio-temporale, per somministrare una prova di calcolo a mente, di organizzazione seriale di cifre, il test del disegno di un orologio o della piantina della camera.

D - RIEVOCAZIONE DIFFERITA

Dopo la prova di interferenza, domandare al soggetto *"Può dirmi le cinque parole che ha letto prima?"*. Per la/le parola/e non ricordata/e (e solo per questa/e) domandare *"Qual era il nome del…?"* fornendo come aiuto il suggerimento semantico corrispondente. Contare il numero delle parole correttamente rievocate (spontaneamente o con aiuto): ***PUNTEGGIO DI RIEVOCAZIONE DIFFERITA (RD)***.

PUNTEGGIO TOTALE = *PUNTEGGIO RIEVOCAZIONE IMMEDIATA + PUNTEGGIO DI RIEVOCAZIONE DIFFERITA*

FOGLIO DI NOTAZIONE -1- RIEVOCAZIONE IMMEDIATA

ITEM	CUE/AIUTO SEMANTICO	RIEVOCAZIONE IMMEDIATA (RI)	
		RI spontanea	RI con cue/aiuto
MUSEO	Edificio		
LUCCIOLA	Insetto		
ARANCIATA	Bevanda		
CAMION	Veicolo		
IMBUTO	Utensile da cucina		

PUNTEGGIO DI RIEVOCAZIONE IMMEDIATA= /5

FOGLIO DI NOTAZIONE -2- RIEVOCAZIONE DIFFERITA

ITEM	CUE/AIUTO SEMANTICO	RIEVOCAZIONE DIFFERITA (RD)	
		RD spontanea	RD con cue/aiuto
MUSEO	Edificio		
LUCCIOLA	Insetto		
ARANCIATA	Bevanda		
CAMION	Veicolo		
IMBUTO	Utensile da cucina		

PUNTEGGIO RIEVOCAZIONE DIFFERITA= /5

PUNTEGGIO TOTALE: PUNTEGGIO RI + PUNTEGGIO RD= /10

MUSEO

LUCCIOLA

ARANCIATA

CAMION

IMBUTO
